# A *Mollicutes* Metagenome-Assembled Genome from the Gut of the Pteropod *Limacina rangii*

**DOI:** 10.1128/mra.00752-22

**Published:** 2022-11-16

**Authors:** Zachary T. Pimentel, Patricia S. Thibodeau, Bongkeun Song, Ying Zhang

**Affiliations:** a Department of Cell and Molecular Biology, College of Environment and Life Sciences, University of Rhode Island, Kingston, Rhode Island, USA; b Department of Biological Sciences, Virginia Institute of Marine Science, William & Mary, Gloucester Point, Virginia, USA; Montana State University

## Abstract

A nearly complete genome of an uncultured *Mollicutes* sp. was obtained from the metagenome of the gut of Limacina rangii (open-ocean snail), an important grazer and prey for higher trophic animals along the rapidly warming region of the western Antarctic Peninsula.

## ANNOUNCEMENT

A recent metabarcoding study of Limacina rangii, a dominant grazer among zooplankton and an important prey along the western Antarctic Peninsula (WAP), revealed that *Mollicutes* bacteria are a cosmopolitan and dominant component of the gut microbiome (1, 2). Here, we report a nearly complete metagenome-assembled genome (MAG) of the class *Mollicutes* from the *L. rangii* gut obtained along the WAP, contributing to the growing number of genomic resources available for host-associated marine *Mollicutes* (3–8).

*L. rangii* organisms were collected at selected stations along the WAP (600.200, 300.200, and 100.040) in January 2017 (9). Gut samples were dissected onboard and immediately frozen at −80°C. Total DNA was extracted from three gut samples per station (9), using the Qiagen DNeasy blood and tissue kit, and then pooled to create a single extract for sequencing. Libraries were prepared with 30 to 50 ng DNA per sample using the Nextera DNA sample preparation kit (Illumina).

Sequencing was performed at a read length of 2 × 150 bp by MR DNA (Molecular Research LP) on the Illumina HiSeq 2500 platform. Raw read quality was visualized with FASTQC version 0.111.14 (10). A total of 31,260,228 read pairs across three samples underwent quality filtering with Trimmomatic version 0.38 (11) with the following parameters: minimum length of 90 bp, 4-bp sliding window with an average quality score of 15, and leading/trailing bases with quality scores less than 3 were removed. Quality-filtered reads were coassembled using default parameters with MEGAHIT version 1.1.1 (12). The coassembly was indexed with bowtie2 version 2.2.9, and the quality-filtered reads from each sample were mapped to the coassembly (13). SAM files were converted to BAM format and ordered using SAMtools release 1.5 (14). MetaBAT2 version 2.12.1 (15) was used to bin the coassembled contigs with default parameters. The *lineage_wf* function in CheckM version 1.0.5 (16) estimated completeness and contamination. Gene prediction and annotation were completed with PGAP (17). Conserved single-copy genes (CSCGs) were identified through analysis of bidirectional best BLAST hits between the MAG, *Mycoplasma* reference genomes, and outgroup *Firmicutes* genomes. Each CSCG cluster was aligned with MUSCLE version 3.8.3, and a phylogenetic reconstruction was performed with RAxML version 8.2.10 using the JTT substitution model and the GAMMA model of rate heterogeneity as previously described (3).

**FIG 1:**
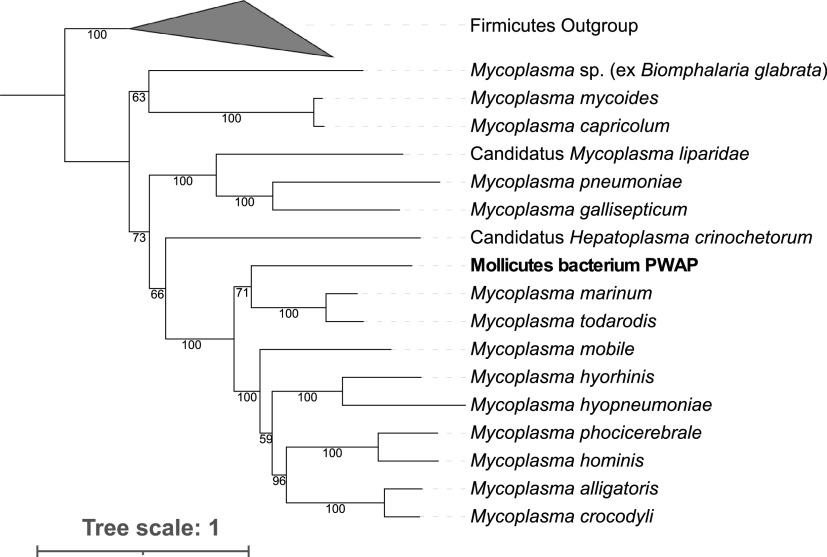
Maximum-likelihood phylogeny based on conserved single-copy genes between the *Mollicutes* MAG from this study (PWAP), reference *Mycoplasma* genomes, and four genomes from the *Firmicutes* used as an outgroup. One hundred iterations were used to compute bootstrap values. The GenBank accession numbers for the genomes in the phylogenetic reconstruction are included in Table 1.

In total, 637,502 contigs were coassembled, but only one MAG, taxonomically assigned to the class *Mollicutes*, was obtained with high estimated completeness. The mean depth of coverage was highest at the most northern site (600.200) at 22.9×, while it was less than 2× at the other sites (300.200 and 100.040). The *Mollicutes* MAG contained 85 contigs and a genome size of 0.55 Mb, with an *N*_50_ of 7,347 bp, estimated completeness of 88.16%, contamination and strain heterogeneity of 0%, and GC content of 25.1%. The *Mollicutes* MAG included 527 protein-coding genes and contained multiple rRNA genes (one each of 16S, 23S, and 5S). The MAG was most closely related to Mycoplasma marinum and Mycoplasma todarodis, which were isolated from an octopus and squid, respectively, based on a phylogenetic reconstruction from 63 CSCGs (Fig. 1; Table 1).

**TABLE 1:** NCBI accession numbers for bacterial genomes included in the phylogenetic reconstruction

Organism	NCBI RefSeq accession no.
Mycoplasma pneumoniae M129	GCF_000027345.1
Mycoplasma gallisepticum	GCF_001676495.1
“Candidatus *Mycoplasma liparidae*”	GCA_009884515.1
*Mycoplasma* sp. (ex Biomphalaria glabrata)	GCF_001484045.1
Mycoplasma hyopneumoniae	GCF_002257505.1
“Candidatus *Hepatoplasma crinochetorum*” Av	GCF_000582535.1
Mycoplasma mycoides subsp. *capri*	GCF_900489525.1
Mycoplasma alligatoris A21JP2	GCF_000178375.1
Mycoplasma capricolum subsp. *capripneumoniae* 87001	GCF_000835085.1
Mycoplasma crocodyli MP145	GCF_000025845.1
Mycoplasma hyorhinis	GCF_001705605.1
Mycoplasma hominis	GCF_000759375.2
Mycoplasma phocicerebrale	GCF_003383595.3
Mycoplasma todarodis	GCF_004335995.1
Mycoplasma mobile 163K	GCF_000008365.1
Mycoplasma marinum	GCF_004335975.1
Lactobacillus plantarum WCFS1	GCF_000203855.3
Listeria monocytogenes EGD-e	GCF_000196035.1
Enterococcus faecalis V583	GCF_000007785.1
Staphylococcus aureus subsp. *aureus* NCTC 8325	GCF_000013425.1

### Data availability.

The raw reads were deposited in the NCBI SRA database with accession numbers SRR12228976, SRR12228977, and SRR12228978, and the MAG assembly is available under ASM1966174v1, all attached to BioProject PRJNA646234.
